# A randomized trial investigating the impact of response expectancy on the counting blessings intervention: the role of optimism as a moderator

**DOI:** 10.3389/fpsyg.2024.1399425

**Published:** 2024-12-03

**Authors:** Petronela Predatu, Daniel David, Irving Kirsch, Ionuț Stelian Florean, Răzvan Predatu

**Affiliations:** ^1^International Institute for the Advanced Studies of Psychotherapy and Applied Mental Health, Babeș-Bolyai University, Cluj-Napoca, Romania; ^2^Evidence Based Psychological Assessment and Interventions Doctoral School, Babeș-Bolyai University, Cluj-Napoca, Romania; ^3^Department of Clinical Psychology and Psychotherapy, Babeş-Bolyai University, Cluj-Napoca, Romania; ^4^Icahn School of Medicine at Mount Sinai, New York, NY, United States; ^5^Program in Placebo Studies, Beth Israel Deaconess Medical Center, Harvard Medical School, Boston, MA, United States

**Keywords:** expectancy, psychological intervention, gratitude, emotions, optimism, randomized controlled trial

## Abstract

**Introduction:**

This randomized controlled trial aimed to address a knowledge gap concerning the mechanisms responsible for the efficacy of gratitude interventions. Specifically, we investigated how various response expectancies (positive, ambiguous + negative, and no expectancy) impact the efficacy of the “counting blessings” intervention in influencing positive and negative emotions. Additionally, the study explores how optimism levels (high, medium, low) interact with these expectancies to influence intervention efficacy.

**Method:**

A total of 529 adult volunteers were recruited through social media and randomly assigned to one of three experimental conditions, Positive Condition (PC), Ambiguous + Negative Condition (ANC), No Expectancy Condition (NEC), using a Random Sequence Generator. Of these, 142 participants completed the seven-day counting blessings intervention, and 111 participated in a follow-up assessment 1 month later. Missing data were addressed using multiple imputation. The main outcomes were changes in positive and negative emotions, with moderation analysis assessing the interaction between optimism levels and response expectancies. The study adhered to the CONSORT guidelines.

**Results:**

While no significant interaction was found between experimental conditions and time regarding emotional outcomes (*p* ˃ 0.05), moderation analysis revealed differential interactions between optimism levels and expectancies, particularly influencing positive emotions (*p* < 0.009). For participants with low optimism, positive emotions significantly increased from post-intervention to follow-up in the PC (*t* = −2.42, *p* < 0.016) and from pre-intervention to post-intervention in the ANC (*t* = 2.41, *p* < 0.018). Participants with medium optimism experienced an increase in positive emotions across all conditions from pre-intervention to follow-up and from post-intervention to follow-up (*p_s_* < 0.05). High optimism participants showed an increase in positive emotions from pre-intervention to follow-up and post-intervention to follow-up in the PC (*t* = 2.09, *p* < 0.038 and *t* = 3.06, *p* < 0.003) and NEC c (*t* = −2.76, *p* < 0.006 and *t* = 2.74, *p* < 0.007).

**Conclusion:**

Our findings emphasize the effectiveness of a brief gratitude journal and underscore the nuanced role of response expectancy, especially in interaction with the initial level of optimism, in enhancing positive emotions. These results hold significance for both theoretical understanding and clinical applications.

## Introduction

Gratitude interventions refer to deliberate practices or exercises aimed at cultivating and enhancing feelings of gratitude. These interventions are known for their simplicity, ease of implementation, and promising outcomes, even within clinical settings. One of the most widely used strategies is the “Three Good Things” (TGT) list. This exercise asks participants to reflect, write about, and explain three good things that occurred during the day and for which they felt grateful (Emmons and McCullough, 2003). Research suggests that this simple and quick activity has various positive outcomes, including increased positive affect, happiness, life satisfaction, well-being, improved pro-social behavior, better sleep, and enhanced concentration (Chopik et al., 2019; Cunha et al., 2019; Mongrain and Anselmo-Matthews, 2012; Salces-Cubero et al., 2019; Watkins et al., 2015; Yang et al., 2018). Moreover, the TGT list appears to be effective in reducing negative affect, depressive symptoms, and loneliness (Bartlett and Arpin, 2019; Dickens, 2019; Salces-Cubero et al., 2019; Southwell, 2012; Yang et al., 2018). While these outcomes are promising, there exists a knowledge gap regarding the mechanisms through which gratitude interventions contribute to the promotion of well-being. Understanding mechanisms of change is essential for improving treatment specificity (Alkozei et al., 2018; Kazdin, 2007). Recent research suggests that gratitude interventions may promote well-being by fostering social connectedness (Alkozei et al., 2018; O’Connell et al., 2018) and promoting positive cognitive processes, such as focusing on and recalling positive experiences (Alkozei et al., 2018). Additionally, gratitude interventions may increase resilience and reduce repetitive negative thinking (Heckendorf et al., 2019), with other proposed mediators including gratitude state, meaningful goal pursuit, and reward processing (Bohlmeijer et al., 2022; Oltean et al., 2022; Otto et al., 2016).

## Response expectancy

According to Kirsch and Lynn (1999), response expectancies are defined as “anticipations of automatic subjective and behavioral responses to particular situational cues, and their effects are a form of self-fulfilling prophecy” (p. 504). Response expectancies are deemed sufficient to determine a nonvolitional outcome, not mediated by other psychological factors, and are self-confirming (Kirsch, 1985). Numerous studies have underscored the significance of response expectancies as a psychological mechanism involved in generating nonvolitional outcomes across various contexts, including psychological treatment, medical interventions, or pharmacological agents (Akroyd et al., 2020; Kirsch, 2019; Kirsch et al., 2015; Rutherford et al., 2017).

The hypothesis that expectancies might be responsible for positive effects in the case of gratitude interventions is not new. Geraghty (2010) conducted a series of studies investigating the role of response expectancy in a gratitude strategy that yielded favorable results. Notably, he illustrated that greater response expectancy for positive affect was directly related to reports of both greater positive affect and less negative affect. Moreover, more and more results have accumulated, arguing that the well-being improvements associated with gratitude interventions may result from this specific process, response expectancy (Davis et al., 2016; Wood et al., 2010). In this regard, Jans-Beken et al. (2020) exploring various gratitude interventions, concluded that delivering distinct instructions to participants—essentially manipulating response expectancies during the same exercise—can yield different outcomes. Therefore, modifying expectations about an intervention not only serves as a way to explore potential mechanisms behind changes in outcomes but also provides an opportunity to optimize interventions. In this context, understanding whether intervention instructions (i.e., manipulating response expectancy) impact the effectiveness of gratitude interventions is a critical direction for further research (Cregg and Cheavens, 2021). Building upon these results, we propose to enhance our understanding of the extent to which response expectancy is indeed the mechanism of change in gratitude techniques and its impact on positive outcomes. Research has generally focused on how verbal suggestions influence expectations by examining several key aspects: the *direction* of the expectation (i.e., whether the treatment is expected to increase or decrease symptoms) (Crichton et al., 2013; Devlin et al., 2019; Kam-Hansen et al., 2014; Kaptchuk et al., 2008), the *magnitude* of the expected effect (i.e., the perceived strength or intensity of the treatment’s impact) (Colloca and Benedetti, 2006; Faasse et al., 2019; Pollo et al., 2001), and more recently, the *temporality* of the expectation (i.e., the anticipated timing of when the treatment effects will manifest) (Camerone et al., 2021; Klinger et al., 2017).

Regarding the direction of manipulated expectancies, they were categorized into three distinct types: positive, negative, and ambiguous or mixed. Research has demonstrated that positive expectancies typically lead to favorable outcomes, regardless of whether the treatment is active or inactive. Conversely, negative expectancies are linked to less favorable outcomes (Crichton et al., 2013; Henrich et al., 2023; Kirsch et al., 2014; Peerdeman et al., 2016; Petrie and Rief, 2019). In placebo literature, the “ambiguous condition” typically refers to a scenario where participants are informed that there is an equal (50%) chance of receiving either an active treatment or a placebo (e.g., Pollo et al., 2001; Kam-Hansen et al., 2014). This approach creates uncertainty about the treatment they will receive and, consequently, about the likelihood of experiencing any beneficial effects from the intervention. In other cases, the “ambiguous condition” does not focus on the uncertainty of receiving an active treatment but rather on the uncertainty regarding the direction of the treatment’s effect. This approach is particularly useful in exploring how unclear or mixed expectations can shape individuals’ responses to treatment.

Kirsch (2018) proposed that response expectancies have two key dimensions: the expected magnitude of the response (the intensity or size of the outcome) and the strength of the expectancy (the subjective probability of a change occurring), though both are rarely assessed together. Furthermore, Kirsch (2018) claimed that response expectancies are fluid and can change with new experiences, emphasizing the importance of repeated measurements within a study.

Moreover, research has indicated that response expectancies and the disposition of optimism-pessimism interact in shaping emotional reactions (Geers et al., 2005, 2007; Geers and Lassiter, 2002; Kern et al., 2020). Optimism-pessimism significantly moderates the relationship between response expectancies and affective experiences. Geers and Lassiter (2002) demonstrated that specific expectations interact with optimism-pessimism based on the consistency between the expectation and stimulus information (positive vs. negative valence). Optimists tend to assimilate both positive and negative experiences into their pre-existing expectations, regardless of discrepancies. These effects persist whether or not the experiences align with their expectations. In contrast, pessimists assimilate only experiences consistent with their expectations (e.g., positive experiences with positive expectations and negative experiences with negative expectations). When expectations and experiences diverge, pessimists highlight the discrepancies. This interaction underscores the complex interplay between optimism-pessimism, response expectancies, and emotional responses.

### Main objective

The primary objective of this research was to fill a gap in understanding the efficacy of gratitude interventions by examining the role of response expectancy within a randomized controlled trial (RCT). Specifically, we aimed to determine whether different types of instructions (positive, ambiguous + negative, or no expectancy) for a gratitude journal would lead to differences in emotional outcomes, both positive and negative.

The study, conducted online over seven consecutive days, is likely to attract participants who are highly motivated and interested in self-help interventions (e.g., Geraghty, 2010; Waits, 2017). In this context, participants may interpret ambiguous information about the potential “mixed” effects of the intervention in a positive and favorable way. Consequently, the distinction between the positive condition and the mixed condition could be too subtle to create a noticeable difference in outcomes. Additionally, providing explicit and strongly negative expectations may be challenging in an online format, where high dropout rates are a well-known concern (e.g., Cunha et al., 2019; Fekete and Deichert, 2022). Given these considerations, we opted for a more nuanced approach in which participants received ambiguous information about the intervention’s effects, coupled with a rationale suggesting potential negative outcomes, such as a reduction in positive emotions and an increase in negative ones. This approach strikes a balance between maintaining credibility and participant engagement while allowing us to explore how ambiguity and negativity interact to shape emotional responses.

Our first hypothesis posited that individuals in the Positive Condition (PC) would report higher levels of positive and lower levels of negative emotions post-intervention and follow-up compared to individuals in the Ambiguous + Negative Condition (ANC) and No Expectancy Condition (NEC). Our second hypothesis suggested that individuals in the ANC condition would report lower levels of positive and higher levels of negative emotions post-intervention and follow-up compared to individuals in the NEC.

### Secondary objectives

The secondary objective of this research was to explore the interaction between optimism levels (low, medium, and high) and specific expectations (positive, ambiguous + negative, and no manipulated expectancy) in relation to the efficacy of gratitude journals.

While the literature on the relationship between optimism and responses to positive or negative expectations has begun to be documented, there is limited research on optimism in relation to ambiguous expectations. To our knowledge, no study has concurrently investigated the effects of optimism levels on both ambiguous and negative expectations within the same experimental condition. Most existing research has focused separately on either positive or negative expectations, with little attention given to how optimism may influence responses when expectations are ambiguous.

Research on dispositional optimism and coping strategies suggests that individuals with lower levels of optimism are more likely to interpret uncertain or ambiguous information negatively compared to those with higher levels of optimism. For instance, studies have shown that lower optimism is associated with a heightened sensitivity to potential threats in ambiguous situations, resulting to more negative interpretations and stronger emotional reactions (Carver and Scheier, 2014; Nes and Segerstrom, 2006). On the other hand, the findings from empirical studies on how individuals with low optimism respond to specific negative expectations are mixed. Some evidence suggests that pessimistic individuals who are given negative expectations tend to report negative effects from the treatment (Geers et al., 2005; Corsi et al., 2016). However, other studies indicate that these individuals may experience an increase in positive affect due to a contrast effect, where the discrepancy between their negative expectations and the actual positive experience leads to an enhanced appreciation of the positive aspects (Geers and Lassiter, 2002). Given the mixed and limited findings, we did not formulate a specific hypothesis regarding the interaction effect between optimism levels in individuals exposed to both ambiguous and negative expectations (ANC). Moreover, previous research has not analyzed how moderate levels of optimism influence emotional responses within these intervention conditions. Therefore, this study adopts an exploratory approach to address this gap in the literature.

### Exploratory objectives

Ultimately, we propose to explore the interplay between levels of optimism and the various facets of response expectancies, encompassing both expected magnitude and strength expectancy for positive and negative emotions, assessed at two distinct time points (response expectancies from Day 1 and response expectancies from Days 2 to 7). We will examine the initial response expectancies, where information regarding intervention efficacy is presented without interaction with the technique (Day 1). On the other hand, the second set of response expectancies characterizes the situation where information and interaction/experience with the technique co-occur (Days 2 to 7, with a mean score calculated). This innovative approach promises valuable insights into the dynamic evolution of two different types of response expectancies in conjunction with a gratitude technique.

## Methods

### Participants

The necessary sample size was determined using the statistical software G*Power (Faul et al., 2007). The specified analysis was a mixed ANOVA (within-between interaction) with three time points and three groups. We assumed a correlation among repeated measures of r = 0.5, an expected effect size of small to medium (Cohen’s *f* = 0.10), a statistical power of 0.80, and a significance level of 0.05. The recommended sample size was 204 participants. The dropout rate was anticipated to be around 50%.

Inclusion criteria required participants to be (a) aged 18 or older and (b) possess a smartphone with regular internet access to enable completion of the intervention. Eligible participants were randomly assigned to one of three conditions using simple randomization with a 1:1:1 allocation ratio, generated through a computerised Random Sequence Generator. The first author (P.P) generated the random allocation sequence, enrolled participants, and assigned them to their corresponding intervention condition. A total of 529 adult volunteers were recruited via social media between June and August 2020. The sample consisted of 462 females (87.3%) and 67 males (12.7%), with an age range of 18 to 63 years (M = 25.44, SD = 8.36). The sociodemographic and clinical characteristics of each group are presented in Table 1. The study was conducted entirely online, with the intervention delivered through a mobile application over seven consecutive days, allowing participants to complete the gratitude exercise remotely. Informed consent was obtained from all participants, and data protection was ensured. The study was approved by the Institutional Review Board of Babeș-Bolyai University.

**Table 1:** Demographic and clinical characteristics.

	Group 1—*Positive expectancies* (*n* = 176)		Group 2—*Ambiguous + Negative expectancies* (*n* = 180)		Group 3—*No expectancy condition* (*n* = 173)		F test	*p*
Age (M/SD)	26.39	9.28	24.45	7.04	25.50	8.56	2.43	0.089
Female (%)	90.03%		85.6%		86.1%		*ꭓ*^2^ = 2.18	0.336
***Status***					
Students (%)	51.7%		57.2%		56.1%			
Employed (%)	38.1%		30.06%		30.6%		*ꭓ*^2^ = 3.21	0.519
Other (%)	10.2%		12.3%		13.3%			
***Clinical characteristics***					
Depression (M/SD)	8.05	5.56	7.76	5.15	7.56	5.26	0.368	0.695
Stress (M/SD)	11.38	5.34	10.96	5.00	10.60	5.83	0.911	0.403
Anxiety (M/SD)	8.18	5.71	7.78	5.14	7.50	5.32	0.708	0.493

Out of the initial 529 participants who expressed their willingness to participate and completed demographics, optimism levels, and measures of negative and positive emotions, as well as depression, anxiety, and stress at T1, only 151 participants (28.54%) actively engaged in the “counting blessings” intervention over the 7-day period. These participants completed the task and reported their daily levels of response expectancies. Subsequently, only 142 participants (26.84%) proceeded to complete the post-intervention measures at T2, specifically reporting their levels of negative and positive emotions, along with their assessment of the credibility of the intervention’s rationale. Finally, 111 participants (20.98%) completed the follow-up assessment (T3), reporting their levels of negative and positive emotions. Participants who successfully completed all stages were eligible to enter a drawing for the chance to win one of three vouchers worth 20 euros, one of three self-development books, or course credit for psychology students.

### Measures

#### Positive and negative emotions

This study employed the Positive Affect and Negative Affect Schedules (PANAS, Watson et al., 1988) to assess emotions. The scale comprises 20 self-report mood-adjectives, divided into two categories: half of the items measure positive affect (PA), such as “inspired,” and the remaining half measure negative affect (NA), such as “distressed.” Scores on the scale can range from 10 to 50 for both positive and negative affect, with higher scores indicating higher levels of PA or NA, respectively. Participants used a 5-point Likert scale to indicate the extent to which they felt specific emotions last week (1 = very slightly or not at all, 5 = extremely). The subscales for PA and NA used in the present study demonstrated good internal consistency (*α* = 0.764–0.903) and (α = 0.683–0.892), respectively.

#### Response expectancies

In this study, participants’ response expectancies were assessed using a single item for each dimension (expected magnitude and strength of the expected) regarding anticipated positive and negative emotions, referring to a specific time when the outcome will occur (e.g., after experiencing this technique—Day 1 to Day 7). The magnitude of the expected response was evaluated with the question (e.g., “How many positive emotions do you expect to feel after this technique?”), while the strength or certainty dimension was assessed differently (“How certain are you that you will feel positive emotion after this technique?”). Participants provided responses on a 5-point Likert scale (1 = not at all, 5 = to a great extent), where higher scores indicated a higher level of response expectancy for each dimension. It is worth noting that research has demonstrated that single-item measurements are valid, reliable, and appropriate for unidimensional constructs (Allen et al., 2022; Ang and Eisend, 2018; Fuchs and Diamantopoulos, 2009).

#### Optimism

The Life Orientation Test-Revised (LOT-R, Scheier et al., 1994), consisting of ten self-report questions (including four filter items), was employed to assess dispositional optimism. The remaining six items are categorized as positively or negatively worded. The LOT-R was treated as a unidimensional construct (Cano-García et al., 2015; Hinz et al., 2021). Optimism and reversed pessimism scores were summed to generate the final score, which ranges from 0 to 24, with higher scores indicating greater optimism levels. The scale has demonstrated good psychometric properties in both general and clinical populations (Hinz et al., 2021; Steca et al., 2017). In this study, the internal consistency of optimism was found to be good (*α* = 0.784). Expanding on the existing research that views optimism as a unidimensional construct (Cano-García et al., 2015; Hinz et al., 2021), we propose to explore this disposition on three different levels (high, medium, and low levels of optimism) to offer a more nuanced perspective.

#### Treatment credibility

Treatment credibility was assessed using the Credibility/Expectancy Questionnaire (CEQ, Devilly and Borkovec, 2000), administered post-intervention. The credibility subscale, which measures logicality, perceived success in reducing negative symptoms, and recommendability to a friend, was used in the current study and adapted for the counting blessings intervention. We introduced an additional item to the credibility subscale to balance the perceived success of the intervention on increasing positive emotions. The current study’s credibility subscale demonstrated good internal consistency (α = 0.934) (see Appendix).

### Procedure

**Figure 1:**
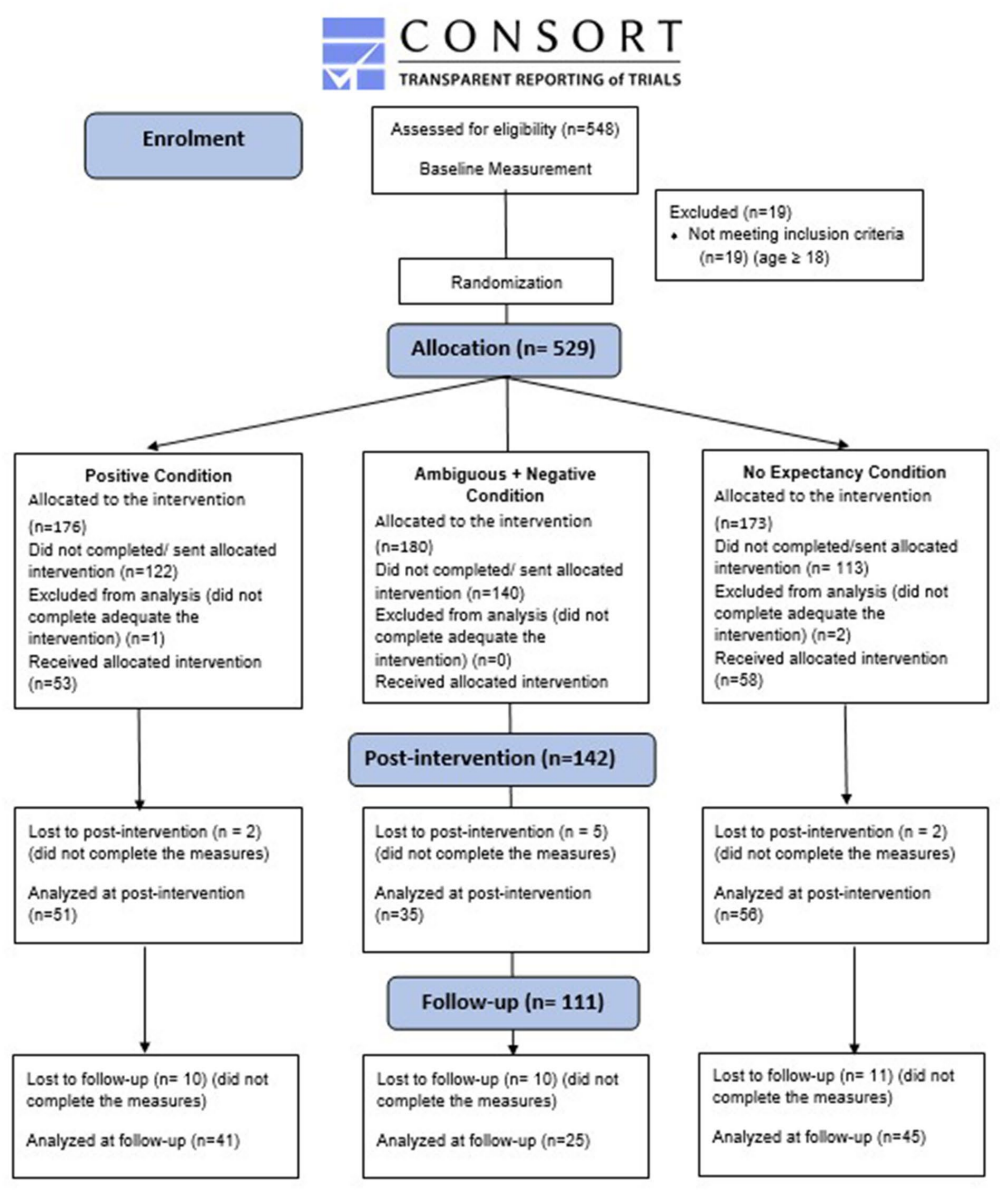
Flow diagram of participant recruitment, allocation, and data collection.

Figure 1 illustrates the recruiting method and data collection in the study, as shown in the flowchart diagram.

**Figure 2:**
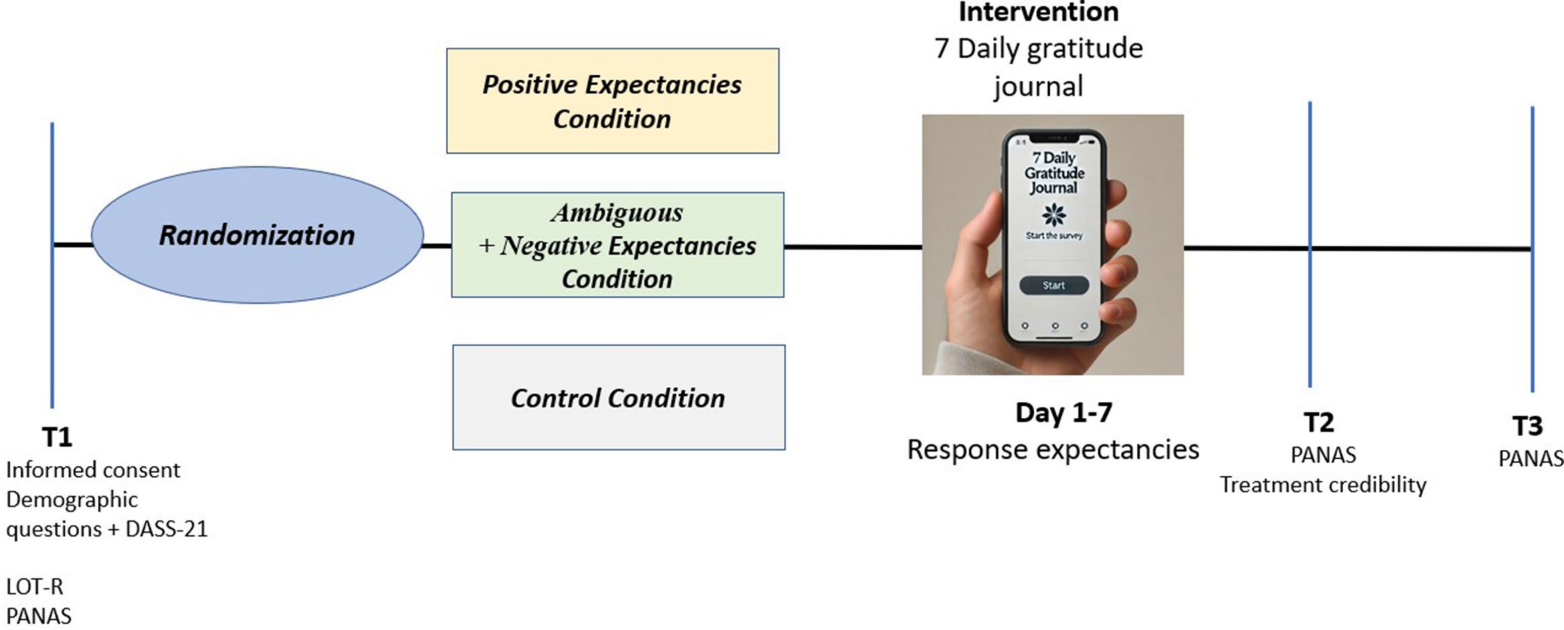
Procedure.

After providing informed consent to participate in the current study, participants completed online demographic data and baseline measures (T1). A total of 529 participants were randomly assigned to one of the experimental groups or the control condition. In the subsequent stage, participants received an email containing instructions for installing the PIEL Survey Application on their mobile devices and the intervention document. Participants were encouraged to reach out to the first author (P.P) in case of technical issues. The intervention was delivered for 7 consecutive evenings, with an automatic notification at 21:30 and two additional notifications in case of non-completion. Each survey included (a) manipulation instructions depending on the condition (see Table 2), (b) questions about response expectancies, and (c) the “counting blessings” technique (see Table 2). In this study, written information manipulated the subjective probability of response expectancy, leading some participants to believe they received an intervention with either a positive, negative, or unclear effect. The estimated time required to complete the intervention was approximately 10 min. The day after concluding the intervention (when the PIEL Survey with the gratitude journal was sent), participants received an email containing a link to complete the post-intervention measures (T2). Follow-up measures were collected a month later (T3). The study was conducted in accordance with the principles outlined in the Declaration of Helsinki (World Medical Association, 2013) A schematic representation of the procedure is shown in Figure 2.

**Table 2:** Manipulation expectations and instruction for TGT.

**Instructions for expectancies manipulation**	***Positive expectancies***: (Positive Condition - Group 1)	Next, we invite you to go through a psychological intervention, based on the technique "count the positive aspects of your life". In terms of its efficacy, results in the empirical literature proved this intervention to be effective. Numerous studies have shown that the recall of good things and positive experiences in the past leads to a significant increase in positive emotions (contentment, gratitude, happiness and satisfaction with life), as well as to adaptive behaviors such as prosocial and problem-solving behaviors. At the same time, the intervention proved to be effective in reducing dysfunctional negative emotions in the form of depression and anxiety, as well as psychopathology in general. The positive results of the intervention were confirmed on various samples consisting of adolescents, adults and the elderly.	***Ambiguous* + *Negative expectancies:*** (Ambiguous + Negative Condition - Group 2)	Next, we invite you to go through a psychological intervention, based on the technique "count the positive aspects of your life ". In terms of its efficacy, results in the empirical literature proved this intervention to have mixed effects. A series of studies have shown that, although this technique can have a beneficial effect in some instances, an increased focus on good things and positive experiences from the past can lead to an opposite, detrimental effect. In this case, a rebound effect causes an increase in negative emotions (dissatisfaction with the present condition, depression, regret, dissatisfaction with life), as well as maladaptive behaviors such as isolation and procrastination. The mixed results of the intervention were confirmed on various samples consisting of adolescents, adults and the elderly.	Nonmanipulated expectancies –(No expectancy condition -Group 3)	Next, we invite you to go through a psychological technique.
	**The “counting blessings” intervention**	The instruction was similar with the intervention proposed by Emmons and Stern (2013), respectively Emmons and McCullough (2003), for all conditions.	“There are many things in our lives, both large and small, that we can be grateful about. We want to focus for a moment on benefits or gifts that you have received in your life. These gifts could be simple everyday pleasures, people in your life, personal strengths or talents, moments of natural beauty, or gestures of kindness from others. We might not normally think about these things as gifts, but that is how we want you to think about them. Take a moment to really savour or relish these gifts, think about their value, and then write them down. every night before going to sleep. You cannot list the same thing more than once on the same day, but you can write the same thing on different days. On the last day, think of 3 things in your life for which you are thankful or grateful, and indicate a causal explanation for each of the 3 good things listed above.”

#### Study conditions and expectancy manipulation

The expectancies were manipulated by written instructions provided prior to the completion of each electronic journal, corresponding to each experimental condition:

#### Positive condition (PC)

In this condition, clear and positively valenced expectations were manipulated to align with the intended effect of the intervention. Participants in the positive condition expected that journaling about good things would have a favourable impact on their emotions, leading to an increase in positive emotions and a decrease in negative ones.

#### Ambiguous + negative condition (ANC)

In this condition, ambiguous and negatively valanced expectations were manipulated to counteract the intended effect of the intervention. Participants in the ambiguous + negative condition expected that journaling about good things would have a mixed impact on their emotions. Although uncertain about the outcome, they leaned toward the belief that the exercise might increase negative emotions and decrease positive ones. This uncertainty, combined with a negative bias, led to scepticism about the effectiveness of journaling. They were concerned that focusing on positive experiences might instead emphasize what they lack or highlight the gap between their current state and those positive moments.

Recognizing the challenge of inducing purely negative expectations toward a positive psychological intervention we used a combination of ambiguous and negative information. This approach has several rationales. First, purely negative expectations toward an intervention that is generally perceived as pleasant might seem unrealistic or implausible to participants, especially if they know the intervention is typically beneficial. By combining ambiguous and negative information, we maintain credibility, as ambiguity reflects the natural uncertainty that often accompanies new experiences, making the expectations more relatable and believable. Second, purely negative expectations can lead participants to resist or reject the information, particularly if they believe in the inherent positivity of the intervention. Introducing a degree of ambiguity creates space for doubt, reducing the likelihood of defensive reactions and increasing the chances of participants engaging with the intervention, which is delivered online over seven consecutive days. Third, this approach allows us to explore how different elements of expectancy (ambiguity and negativity) interact to influence emotional outcomes. It can reveal how ambiguity interacts with a negative bias to shape participant’s experiences and the overall effectiveness of the intervention.

#### No expectancy condition (NEC)

In this condition, no specific expectations regarding the direction of the intervention’s effect were manipulated.

Although the information provided and expectations varied across the three conditions, all participants received the same psychological treatment. The gratitude intervention in this study was based on the ‘Three Good Things’ list developed by Emmons and McCullough (2003), and Emmons and Stern (2013), with identical instructions provided for all conditions.

This study adhered to the CONSORT guidelines for reporting randomized controlled trials (Schulz et al., 2010), with the checklist provided in Supplementary material 1. A list of definitions for the abbreviations used is also provided in Supplementary material 2.

### Data analysis plan

Descriptive statistics were computed, plotted, and visualized in SPSS (George and Mallery, 2019). All these statistics were computed on complete case data. Hypotheses were tested, implying linear mixed models (with random intercepts) in RStudio (RStudio Team, 2019). As for the three measurements (T1, before intervention; T2, after the intervention; T3; follow up), the percentage of missing data was high; we used multiple imputations by chained equation (MICE) to deal with missing data (Van Buuren and Groothuis-Oudshoorn, 2011). The MICE method has several advantages. Missing data are imputed with the most probable values given the available data. This process is repeated several times (in our case, 100 times), and each time a slightly different complete dataset is generated on which the analysis is performed (as if the dataset were complete). The final step involves combining the statistics obtained across the imputations. In our case, this was done across the 100 sets using Rubin’s rule (Rubin, 1987). Thus, MICE accounts for the uncertainty inherent in missing values by generating several possible datasets and maintaining statistical power (Van Buuren and Groothuis-Oudshoorn, 2011; Peterson and Martin, 2017). We considered missing data for those who did not send us the intervention journal survey or did not adequately complete the intervention (see Figure 1). For daily measurements, we used all available cases as per implemented in mixed linear models (Hedeker and Gibbons, 1997). The main R packages used were ‘mitml’ (Grund et al., 2023) and ‘lme4’ (Bates, 2023). We also assessed the moderator role of optimism in the relationship between group and emotional outcomes. Where the three-way interaction effect (group * time * optimism) was found significant we further assessed the condition * time interaction for different levels of optimism. Specifically, as it is convention, we split the participants into three groups based on their optimism level. Low optimism group was composed of those with levels one standard deviation below the mean, average optimism group was composed of those with levels on optimism between −1 and 1 standard deviation. Finally, high optimism group was made up from those who had higher than one standard deviation above the mean. This split was made to probe the moderation effect of optimism. Specifically, we did it to understand how groups change over time at different levels of optimism. All *post hoc* analysis were conducted controlling for type I error with the false discovery rate method (Benjamini and Hochberg, 1995).

## Results

A total of 529 participants were randomly assigned to one of three conditions: Positive Condition (n = 176), Ambiguous + Negative Condition (n = 180), and No Expectancy Condition (n = 173). There were no significant differences between the groups in terms of age (*F* = 2.43, *p* = 0.089), gender distribution (χ^2^ = 2.18, *p* = 0.336), or employment status (χ^2^ = 3.21, *p* = 0.519). Baseline clinical characteristics, including depression (*F* = 0.368, *p* = 0.695), stress (*F* = 0.911, *p* = 0.403), and anxiety (*F* = 0.708, *p* = 0.493), also showed no significant differences between the three groups (see Table 1), indicating successful randomization.

### Manipulation check

The results revealed significant differences between the three groups concerning response expectancy from Day 1 [*F* (2, 148) = 7.349, *p* < 0.001]. Specifically, individuals from the ANC reported more subjective probability for experiencing negative emotions (i.e., expected strength for negative emotions) compared to individuals from the NEC (mean difference = 0.628, SE = 0.16, *p* < 0.001). While a marginally unsignificant difference was observed between individuals from the ANC and PC (mean difference = 0.374, SE = 0.16, *p* = 0.078), with individuals from the ANC showing a trend towards higher levels of subjective probability for experiencing negative emotions compared to individuals from the PC. Thus, manipulating individuals’ response expectancies proved effective in the current study.

### Main analysis—intervention efficacy

#### Positive emotions

No significant main effect existed for *group* for positive emotions, *F* (2, 694) = 0.20, *p* = 0.813. However, a significant main effect of *time* was found, *F* (2, 302) = 16.17, and *p* < 0.001. Finally, no significant *interaction* between condition and time was found, *F* (4, 301) = 0.27 and *p* = 0.893.

#### Negative emotions

No significant main effect existed for *group* for negative emotions, *F* (2, 691) = 1.48, *p* = 0.227. A significant main effect existed for *time*, *F* (2, 296) = 32.14, and *p* < 0.001. Finally, no significant *interaction* between condition and time existed for negative emotions, *F* (4, 295) = 0.143, *p* = 0.965.

Descriptive statistics for positive and negative emotions are presented in Table 3.

**Table 3:** Means and standard deviations for positive and negative emotions.

Variables	Positive condition (*n* = 41)						Ambiguous + negative condition (*n* = 25)						No expectancy condition (*n* = 45)					
	Pre		Post		Follow-up		Pre		Post		Follow-up		Pre		Post		Follow-up	
	*m*	*SD*	*m*	*SD*	*m*	*SD*	*m*	*SD*	*m*	*SD*	*m*	*SD*	*m*	*SD*	*m*	*SD*	*m*	*SD*
**Positive Emotions**	28.48	8.36	29.19	6.04	31.46	8.91	28.27	7.95	30.05	5.76	32.60	7.40	28.97	8.31	29.71	6.17	33.73	8.15
**Negative Emotions**	28.03	8.79	23.47	5.83	23.65	8.24	26.86	8.38	22.60	4.60	21.52	6.81	26.62	9.50	22.92	5.10	22.35	8.07

#### Treatment credibility

To assess perceived credibility, an ANOVA was conducted. There was no significant main effect observed for the group concerning this variable (*p* > 0.05). Therefore, individuals from all conditions reported similar levels of credibility regarding the intervention’s effectiveness in impacting negative and positive emotions post-intervention.

### Secondary analysis—moderation analyses

#### Positive emotions

We found a significant time * group * optimism interaction, with *t* = −2.64 and *p* < 0.009 (see Supplementary Material 3, Table S2).

In the case of low optimism, pairwise comparisons at different moments indicated a significant within-subjects effect (post-intervention vs. follow-up) for PC (*t* = −2.42, *p* < 0.016), respectively, a significant within-subjects effect (pre-intervention vs. post-intervention) for ANC (*t* = 2.41, *p* < 0.018) (see Supplementary Material 3, Table S3). However, no more significant within-subjects effects (pre-intervention vs. post-intervention, pre-intervention vs. follow-up, and post-intervention vs. follow-up) were found for each condition (see Supplementary Material 3, Table S3). Pairwise comparisons revealed that participants with a low optimism from all three conditions did not significantly differ regarding positive emotions at pre-intervention, post-intervention, or follow-up (*p* > 0.05) (see Supplementary Material 3, Table S4).

In the case of medium optimism, pairwise comparisons at a different moment indicated a significant within-subjects effect (pre-intervention vs. follow-up (*t* = 3.73, *p* < 0.001) and post-intervention vs. follow-up (*t* = 3.28, *p* < 0.001)) for PC, a significant within-subjects effect (pre-intervention vs. follow-up (*t* = 3.39, *p* = 0.001), and post-intervention vs. follow-up (*t* = 3.43, *p* = 0.001)) for ANC, respectively a significant within-subjects effects (pre-intervention vs. follow-up (*t* = 3.74, *p* < 0.001), and post-intervention vs. follow-up (*t* = 2.59, *p* < 0.01)) for NEC (see Supplementary Material 3, Table S5). However, for each condition, no significant differences were found between pre-intervention vs. post-intervention (*p* > 0.05) (see Supplementary Material 3, Table S5). Pairwise comparisons revealed that participants with a medium optimism from all three conditions did not significantly differ regarding positive emotions at pre-intervention, post-intervention, or follow-up (*p* > 0.05) (Supplementary Material 3, Table S6).

In the case of high optimism, pairwise comparisons at different moments indicated a significant within-subjects effect (pre-intervention vs. follow-up (*t* = 2.09, *p* < 0.038), and post-intervention vs. follow-up (*t* = 3.06, *p* < 0.003)) for PC, a significant within-subjects effect (pre-intervention vs. post-intervention (*t* = −2.76, *p* < 0.006), and post-intervention vs. follow-up (*t* = 2.74, *p* < 0.007)) for NEC (see Supplementary Material 3, Table S7). However, no more significant within-subjects effects were found for each condition. Pairwise comparisons revealed that participants with a high optimism from all three conditions did not significantly differ between them regarding positive emotions at pre-intervention and post-intervention (*p* > 0.05) (see Supplementary Material 3, Table S7), while at follow-up was obtained a marginal unsignificant effect between PC and ANC (*t* = −1.92, *p* = 0.057)(see Supplementary Material 3, Table S8). The mean and standard deviation for optimism as a moderator and positive emotions as an outcome are presented in Table 4.

**Table 4:** Optimism as a moderator and positive emotions as an outcome.

	Optimism			Positive emotions at T1			Positive emotions at T2			Positive emotions at T3		
	*N*	*m*	*SD*	*N*	*m*	*SD*	*N*	*m*	*SD*	*N*	*m*	*SD*
***Positive Condition***												
High optimism	30	20.80	1.58	30	35.30	8.07	5	37.00	6.04	4	44.50	7.18
Medium optimism	113	13.61	2.77	113	28.08	7.67	35	28.66	5.94	27	33.07	6.31
Low optimism	34	5.29	2.38	34	23.82	7.00	10	27.10	3.69	10	21.90	5.97
***Ambiguous + Negative Condition***												
High optimism	25	20.64	1.52	25	33.52	8.89	6	34.17	6.64	5	35.80	9.06
Medium optimism	134	13.91	2.85	134	28.17	7.19	28	28.93	5.18	19	32.79	5.53
Low optimism	21	5.28	2.28	21	22.61	7.75	1	37.00	–	1	13.00	–
***No expectancy Condition***												
High optimism	34	20.68	1.60	34	33.91	6.49	14	31.79	4.33	11	36.55	4.43
Medium optimism	107	13.26	2.85	107	29.00	7.90	32	30.47	5.77	27	33.78	8.10
Low optimism	32	5.40	2.74	32	23.65	8.30	10	24.40	7.13	7	29.14	11.43

#### Negative emotions

We did not find a significant time * group * optimism interaction, with *t* = 1.50 and *p* = 0.136 (Supplementary Material 3, Table S1).

### Exploratory analysis—*expected magnitude and strength*

#### Positive emotions

Regarding the response expectancies evolution and positive emotions as outcomes, we ran two linear mixed models with random intercepts during the daily measure for the three conditions. We focused on each sub-dimension of response expectancy: Expected Magnitude for Positive Emotions and Expected Strength for Positive Emotions.

##### Expected magnitude for positive emotions

Regarding positive emotions as the outcome, no significant interaction effect was found between group * time * optimism for Expected Magnitude for Positive Emotions, where F (12; 776) = 1.29 and *p* = 0.213.

##### Expected strength for positive emotions

Regarding positive emotions as the outcome, no significant interaction effect was found between group * time * optimism for Expected Strength for Positive Emotions, where F (12; 776) = 0.39 and *p* = 0.965.

#### Negative emotions

Regarding the response expectancies evolution and negative emotions as outcomes, we ran another two linear mixed models with random intercepts during the daily measure for the three conditions. We focused on each sub-dimension of response expectancy: Expected Magnitude for Negative Emotions and Expected Strength for Negative Emotions.

##### Expected magnitude for negative emotions

In the case of negative emotions as the outcome, no significant interaction effect between group * time * optimism for Expected Magnitude for Negative Emotions was found where F (12; 777) = 1.69 and *p* = 0.063.

##### Expected strength for negative emotions

In the case of negative emotions as the outcome, a significant *interaction effect* between group * time * optimism for Expected Strength for Negative Emotions was found, F (12; 776) = 1.94, *p* < 0.026. Therefore, we took an in-depth look at how the conditions, time, and optimism levels interact to influence the Expected Strength for Negative Emotions levels.

In the case of low optimism, pairwise comparisons for the first day (when the intervention is absent) indicated a significant difference between the ANC and NEC (*t* = 2.69, *p* < 0.022). Pairwise comparisons during the intervention (when information and intervention are present) indicated a significant positive difference between the ANC and NEC in the case of Day 3 (*t* = 2.70, *p* < 0.015), Day 4 (*t* = 2.43, *p* < 0.023), Day 6 (*t* = 3.31, *p* < 0.001) and Day 7 (*t* = 3.33, *p* < 0.002), respectively a significant positive difference between the PC and NEC in the case of Day 3 (*t* = 2.58, *p* < 0.015), Day 4 (*t* = 2.58, *p* < 0.023), Day 5 (*t* = 2.67, *p* < 0.023), Day 6 (*t* = 4.55, *p <0.001),* and Day 7 (*t* = 2.91, *p* < 0.005).

In the case of medium optimism, pairwise comparisons for the first day (when the intervention is absent) indicated a significant positive difference between the ANC and NEC (*t* = 3.57, *p* < 0.001), respectively a significant negative difference between the PC and ANC (*t* = −2.40, *p* < 0.025). Pairwise comparisons during the intervention (when information and intervention are present) indicated a significant positive difference between the ANC and NEC in the case of Day 3 (*t* = 2.52, *p* < 0.036), Day 6 (*t* = 2.94, *p* < 0.010), and Day 7 (*t* = 2.60 *p* < 0.029), respectively a significant positive difference between the PC and NEC in the case of Day 6 (*t* = 2.20, *p* < 0.042).

In the case of high optimism, pairwise comparisons for the first day (when the intervention is absent) indicated a marginally unsignificant effect between the ANC and NEC (*t* = 2.10, *p* = 0.058), respectively between the PC and ANC (*t* = −2.07, *p* = 0.058). Pairwise comparisons during the intervention indicated any significant difference between the conditions.

## Discussion

The primary objective of our study was to address knowledge gap related to the mechanisms through which gratitude interventions contribute to the promotion of well-being. In this context, we aimed to assess the influence of response expectancy on the effectiveness of a gratitude intervention among a sample of healthy adults. Specifically, we investigated whether providing positive, ambiguous + negative, or no expectancy instructions for a short daily journal (Three Good Things List) delivered in a mobile digital format would lead to different outcomes, as suggested in the literature (Cregg and Cheavens, 2021; Jans-Beken et al., 2020). Additionally, we aimed to determine whether the level of optimism moderates the relationships between specific expectancies (positive, ambiguous + negative, and no expectancy) and emotional outcomes (positive and negative emotions). Furthermore, we explored the impact of the strength of the response expectancy and the magnitude of the response expectancy on determining emotional outcomes.

The study found no significant differences in emotional outcomes between the conditions post-intervention and at follow-up. However, moderation analysis revealed that optimism levels influenced positive emotions. Participants with low optimism reported increased positive emotions from post-intervention to follow-up in PC and from pre-intervention to post-intervention in the ANC. Participants with medium optimism experienced increased positive emotions across all conditions from pre-intervention to follow-up and post-intervention to follow-up. Participants with high optimism reported increased positive emotions from pre-intervention to follow-up and post-intervention to follow-up in the PC and NEC.

### Response expectancies and intervention efficacy

Contrary to our hypothesis, the groups did not significantly differ in positive or negative emotions reported at post-intervention or follow-up. This suggests that the short daily gratitude list increased positive emotions and reduced negative emotions, regardless of participants’ expectations. The lack of significant results could have three possible explanations. First, the provided instructions may not have been sufficiently strong, credible, and compelling to elicit an effect. Future studies should focus on enhancing the confidence in expected outcomes by using varied modes of information transmission (e.g., verbal, written, video). Although researchers have begun exploring the mechanisms of gratitude interventions, more studies are needed to fully understand how response expectancies influence positive outcomes (Regan et al., 2023; Sheldon and Yu, 2022; Walsh et al., 2023). Another explanation could be that response expectancy impacts outcomes in laboratory settings but not necessarily in real-word contexts, as previous studies suggest (e.g., Geraghty, 2010; Hyland and Whalley, 2008). In ecological contexts, factors such as self-selection, motivation, effort, and person-activity fit, may play a critical role in the effectiveness of self-help online interventions (Waits, 2017). Participants may have already held high positive expectations or intrinsic motivation. Future research should control for these individual characteristics before drawing firm conclusions. The last possible explanation for the obtained findings could be that response expectancy is not the primary mechanism through which the gratitude intervention works, and other specific mechanisms (e.g., reduced repetitive negative thinking) may be responsible for the efficacy of this technique. More research is needed before solid conclusions can be drawn.

### Moderation effects

Empirical findings in positive psychology interventions have shown contradictory evidence concerning the moderating effects of personality traits on well-being. The concordance and resistance hypotheses (McCullough et al., 2004), have been supported from both perspectives. For instance, some research has illustrated that individuals with higher baseline trait gratitude reported greater increases in well-being post-intervention (Dossett, 2011; Hartanto et al., 2019, 2023; Heekerens et al., 2022; Watkins et al., 2003). Conversely, other studies have demonstrated that individuals with lower trait gratitude may benefit more from gratitude interventions (Chan, 2013; Harbaugh and Vasey, 2014; Kurian and Thomas, 2023; Rash et al., 2011).

In the current study, we proposed to examine the moderating effect of optimism on both positive and negative emotions. To gain a more nuanced understanding, we introduced an intermediate variable representing medium optimism. This variable plays a crucial role in interacting with different types of valenced expectations. The moderation analyses yielded significant results, suggesting that various levels of optimism interact differently with participants’ expectations in influencing positive emotions following a positive intervention. This underscores the importance of tailoring interventions to specific populations to maximize their benefits (Dickerhoof, 2007; Froh et al., 2009; Kloos et al., 2022; Waits, 2017).

#### Low optimism

Individuals with low optimism in the PC and ANC benefited significantly immediately after the intervention. Specifically, pessimistic individuals who received specific and clear positive expectations or even ambiguous + negative expectations reported significantly more positive emotions immediately after the intervention than pessimistic individuals in the no expectancy condition. To our knowledge, no prior research has investigated the interaction between different levels of optimism and both ambiguous and negative expectations in shaping emotional outcomes. However, this finding aligns with research indicating that optimism does not always correlate with positive affect, nor does pessimism always correlate with negative affect (Geers and Lassiter, 2002). Geers and Lassiter (2002) demonstrated that specific expectations interact with optimism and pessimism based on the consistency between the expectation and stimulus information (positive vs. negative valence). In their studies, pessimistic individuals with positive or negative expectations for the same funny film clip reported increased positive affect. From this perspective, researchers propose an interactionist approach to explain how optimism-pessimism dispositions interact with specific expectations to influence emotional experiences.

A possible interpretation for our results is that pessimistic individuals in the ANC, who received ambiguous + negative expectancies (incongruent with the actual positive experience), contrasted this discrepancy (e.g., positive experience with negative expectations). This contrast may have prompted them to pay more attention to the inconsistent information (positive experience), potentially leading to greater recognition of positive aspects in the gratitude intervention. Consequently, this could have enhanced positive affect. Similarly, pessimistic individuals in the PC, who received positive expectancy congruent with the actual positive experience, assimilated their affective responses with the positive expectation. This assimilation might have resulted in increased positive affect. Finally, pessimistic individuals in the NEC, who received no specific expectancy, neither contrasted nor assimilated the experience to a previous expectation, potentially resulting in decreased positive affect. However, it is crucial to interpret our findings with caution, especially in the case of low optimism in the ANC, as we had only one participant in this category, preventing us from making any firm conclusions. Additionally, other research suggests that pessimistic individuals in the Negative Condition are more likely to perceive the negative effects of the treatment (nocebo effect) (Geers et al., 2005; Corsi et al., 2016). Before drawing accurate conclusions, replicating our results is essential.

Our findings are consistent with previous meta-analyses (Davis et al., 2016; Dickens, 2017; Wood et al., 2010), suggesting that short gratitude interventions can benefit pessimistic individuals, especially in the short term. To sustain long-term effects, more extended and multi-component gratitude activities may be necessary. These results also align with the resistance hypothesis (McCullough et al., 2004) indicating that individuals with lower levels of a specific positive disposition may benefit from relevant positive interventions. Other studies (Froh et al., 2009; Kurian and Thomas, 2023; Sergeant and Mongrain, 2014) also support the idea that positive interventions are more effective for individuals with lower baseline positivity levels (i.e., low optimism, low dispositional gratitude, low positive affect).

#### Medium optimism

Individuals with moderate optimism across all intervention conditions experienced significant and consistent benefits from the gratitude intervention. They reported increased positive emotions immediately after the intervention, and importantly, this positive effect was maintained a month later, irrespective of the type of expectancy instructions (positive, ambiguous + negative, or no expectancy) they received regarding the intervention’s effectiveness. A possible interpretation for this finding, considering the interactionist perspective involving optimism disposition, specific expectancies, and positive emotional experience, is that when optimism levels are moderate—neither excessively high nor low—specific expectancies may not strongly influence the effectiveness of a positive intervention. This suggests that individuals with moderate levels of optimism may possess a balanced outlook that enables them to derive and sustain emotional benefits from the gratitude intervention, regardless of the type of expectancy set before the intervention. Thus, for these individuals, the positive emotional experience induced by the gratitude intervention appears to be less susceptible to variations in expectancy instructions.

#### High optimism

Individuals with high optimism in both the PC and NEC experienced significant immediate benefits after completing the intervention, and these benefits persisted at follow-up. Specifically, optimistic individuals who received positive or no explicit expectancies reported significantly higher positive emotions post-intervention and a month later than optimistic individuals in the ANC. These findings align with theoretical research, particularly the concordance hypothesis (McCullough et al., 2004) suggesting that individuals with a high positive trait at baseline would substantially benefit from a corresponding positive intervention. For instance, Littman-Ovadia and Nir (2014) demonstrated that a higher initial optimism level enhanced intervention efficacy by reducing negative emotions and increasing positive emotions and optimism.

In the case of optimistic individuals in the ANC, our data indicated a non-significant increase in positive emotions at the end of the intervention and follow-up. A similar pattern was observed in a study by Geers and Lassiter (2002) where optimists exposed to negative expectations for a positive experience reported lower levels of positive affective reactions and less enjoyment of the experience compared to pessimists in the same condition. Interestingly, the same authors suggested that when no expectations were given, optimistic individuals reported more positive affect after an enjoyable film clip than pessimists in the same condition, similar to our results. Given these findings, the influence of response expectancies on emotional outcomes appears evident in the case of optimistic individuals.

### Daily measures

The dynamic interaction between optimism levels and response expectancy dimensions during the intervention phase holds clinical significance. Modifying expectations can occur through various means, such as verbal communication, personal experiences, and observational learning (Henrich et al., 2023). Consistent measurement of response expectancies allows us to capture their fluid nature in response to new experiences, including interactions with the technique itself (Kirsch, 2018). In this context, we examined two specific timeframes. On the first day, expectations were solely based on written information, with different content for each experimental condition. In the intermediate days (when we calculated a mean score for Day 2 to Day 7), expectations were influenced by both written information and personal experience with the technique.

On Day 1, when information about the intervention’s efficacy was presented without prior experience with the technique, pessimistic individuals and individuals with a medium optimism level from the ANC reported a higher subjective probability (strength of the expectancy) of experiencing negative emotions compared to their counterparts in the NEC. Optimistic individuals from the PC indicated a lower subjective probability (strength of the expectancy) of experiencing negative emotions than optimistic individuals from the ANC or NEC. Similarly, individuals with medium optimism from the PC reported a lower subjective probability (strength of the expectancy) of experiencing negative emotions than their counterparts in the ANC. These results underscore the impact of instructions on the efficacy of the counting blessings intervention, interacting with individuals’ optimism levels to influence proximal expectancies regarding the effectiveness of this specific technique (i.e., response expectancy about its effects). In the absence of experience with this particular technique, the interplay between optimism levels and response expectancy for positive emotions appears more relevant in determining positive emotions than the singular contribution of response expectancy alone. Following this logic, in the clinical field, psychotherapists must be sensitive to individuals’ optimism levels before providing an appropriate dosage of positive expectancy to optimize results.

During the intermediate days, when instructions and the experience with the technique co-occurred, individuals with low and medium optimism levels from the ANC maintained a higher subjective probability (strength of the expectancy) of experiencing negative emotions compared to individuals in the NEC. This result partially confirms our hypothesis and suggests that continuous ambiguous + negative expectations had an impact even when the participants had systematic experience with the positive technique, particularly for individuals with low or medium levels of optimism.

An unexpected finding is that pessimistic individuals who received congruent (positive) expectations with the technique valence (positive) in the PC maintained a higher subjective probability (strength of the expectancy) of experiencing negative emotions during the intervention compared to pessimistic individuals in the NEC. Specifically, pessimistic individuals in the PC contrasted their emotional reactions with the given expectations, possibly due to their greater sensitivity to contradictions. This trend aligns with previous literature, which observed a similar pattern in pessimistic individuals who received incongruent (negative) expectations for a positive task (Geers and Lassiter, 2002).

### Strengths

Our findings contribute to the existing literature, suggesting that a short daily gratitude list can be an effective, low-cost, and ecological way to reduce negative affect and increase positive affect for individuals (Fekete and Deichert, 2022).

The inclusion of the “ambiguous + negative” expectancy condition represents a contribution of our study by examining how uncertain and negatively biased expectations influence emotional responses. This approach goes beyond the traditional positive–negative dichotomy, providing insights into how ambiguity and negativity together impact emotional outcomes, which can inform the development of more personalized psychological interventions.

Furthermore, our study significantly contributes to our understanding of expectations. Notably, we directly manipulated expectations for specific experiences, which is crucial since individuals often do not generate their expectations. This situation is analogous to real life, where expectations can be influenced or conveyed by various sources, including friends, strangers, or authoritative figures such as healthcare professionals or experts in specific fields, to individuals. An essential strength of the treatment outcome expectancies measure used in our study lies in its nuanced approach to evaluating participants’ response expectancies through a tailored assessment of both the anticipated magnitude and strength of expected positive and negative emotions at specific time points following the intervention. Importantly, our findings revealed that response expectancy, as a mechanism involved in the efficacy of psychological interventions, remains a complex phenomenon influenced by the rationale of the information provided, with clear implications for the clinical field. Another essential point to note regarding response expectancies is that the emotional outcome (e.g., positive emotions) is not exclusively and directly influenced by congruent response expectancy valence (response expectancy for positive emotions) but also by expectations with the opposite valence (e.g., response expectancy for experiencing negative emotions). The same tendency was confirmed by another study (David et al., 2006), which showed that response expectancies for relaxation (expected magnitude) were inversely related to distress reported prior to the exam. In the end, our findings support the interaction between specific expectations and a person’s level of optimism in influencing emotional responses.

## Limitations and future directions

Findings from this study need to be interpreted in light of several limitations. First, the study recruited a convenience sample through social media and university groups. The majority of participants were healthy individuals from a collectivistic culture with higher levels of well-being, which may limit the generalizability of the results. Future studies should aim to replicate these findings on a more diverse sample, considering cultural and sociodemographic backgrounds and clinical status. Notably, individuals with low or high optimism were underrepresented in our sample, especially at the follow-up measure (e.g., ANC). Second, our sample predominantly consisted of women. Therefore, it is important to replicate these results with gender-balanced samples. Third, the study had a high dropout rate, which is typical of self-guided online interventions. Other internet-based interventions have reported similar attrition rates (e.g., Cunha et al., 2019; Fekete and Deichert, 2022; Geraghty, 2010). Our findings may not be generalizable to laboratory-based research when participants are more motivated by extrinsic factors. Another important limitation of this study is the lack of rigorous control over comparison conditions, which may have impacted the equivalence between experimental groups. Future research should implement more refined manipulations, focusing on either pure positive versus negative expectations or ambiguous combined with positive versus ambiguous combined with negative expectations. Additionally, developing more sophisticated and credible rationales for these expectations would help ensure that participants genuinely believe in them. These improvements would enhance the validity of the findings and provide clearer insights into how different types of expectations influence emotional responses. Furthermore, another limitation of the study is the lack of control over several relevant variables, such as internal factors (e.g., state mood disorders, overall symptom severity, and diagnostic comorbidities) and external factors (e.g., ongoing therapy among participants). Additionally, demographic variables like gender were not controlled, despite evidence suggesting that females tend to have more positive pretreatment or early treatment outcome expectations (Vîslă et al., 2021). Future research should control for these factors to better isolate the effects of the interventions and provide clearer insights into their efficacy across different populations. Finally, another important limitation is that this randomized controlled trial was not pre-registered.

Despite the high dropout rate, those who completed the intervention benefited. Finally, the psychological intervention used in this study may differ significantly from active treatments in pharmacological settings. Consequently, the findings presented here are likely to have greater relevance to psychological outcome variables rather than pharmacological ones.

## Conclusion

Our results add to the body of knowledge, indicating that a brief daily practice of listing things to be grateful for can serve as an efficient, low-cost, and ecological way to decrease negative emotions and enhance positive emotions in healthy individuals. While response expectancy was not found to be the primary mechanism in the effectiveness of gratitude interventions, our findings indicate that some response expectations (e.g., response expectancy for positive emotions) may be more effective than others in achieving the desired outcomes. Furthermore, our findings showed that the intervention effect above positive emotions was moderated by the level of optimism at the beginning of the intervention. In the PC, individuals with high or medium optimism benefited post-intervention and follow-up, while pessimistic individuals benefited only post-intervention. In the ANC, those with medium optimism benefited considerably post-intervention and follow-up. In contrast, for only pessimistic individuals from this condition, the benefit seems to be short-term (post-intervention). For the NEC, only individuals with high or medium optimism benefited significantly from the intervention, while pessimistic individuals did not. Overall, our results suggest that the expectation of positive outcomes (e.g., response expectancy for positive emotions) in some instances can enhance the beneficial effects of a gratitude intervention. These findings can assist researchers and practitioners in creating, customizing, implementing, and developing future gratitude-based interventions.

## Data Availability

Publicly available datasets were analyzed in this study. This data can be found here: https://data.mendeley.com/datasets/w7zrrvn2gb/1.
