# Supplementary material for: A randomized trial investigating the impact of response expectancy on the counting blessings intervention: the role of optimism as a moderator
Source: Front Psychol. 2024 Dec 3;15:1399425. doi: 10.3389/fpsyg.2024.1399425 (PMC11649415; doi:10.3389/fpsyg.2024.1399425)
Supplement: Supplementary file 2 [file Data_Sheet_2.docx]

**Supplementary Materials: List of Abbreviations**

| **Abbreviations** | **Definition** |
| --- | --- |
| ANC | Ambiguous + Negative Condition |
| ANOVA | Analysis of Variance |
| MICE | Multiple imputations by chained equation |
| NA | Negative affect |
| NEC | No Expectancy Condition |
| PA | Positive affect |
| PC | Positive Condition |
| RCT | Randomized Controlled Trial |
| T1, T2, T3 | Time 1, Time 2, Time 3 |
| TGT | Three Good Things |
