# Supplementary material for: A randomized trial investigating the impact of response expectancy on the counting blessings intervention: the role of optimism as a moderator
Source: Front Psychol. 2024 Dec 3;15:1399425. doi: 10.3389/fpsyg.2024.1399425 (PMC11649415; doi:10.3389/fpsyg.2024.1399425)
Supplement: Supplementary file 3 [file Data_Sheet_3.docx]

***Supplementary Material***

| ***Variables*** | ***Estimate*** | ***t*** | ***p*** |
| --- | --- | --- | --- |
| Intercept | 40.73 | 11.15 | < .001 |
| Group | 0.78 | 0.46 | .647 |
| Time | -1.88 | -0.84 | .400 |
| Optimism | -0.73 | -2.87 | .004 |
| Group*Time | -1.46 | -1.39 | .166 |
| Group* Optimism | -0.11 | -0.91 | .363 |
| Time* Optimism | -0.05 | -0.31 | .761 |
| Group*Time* Optimism | 0.11 | 1.50 | .136 |

**Table S1**. The interaction effect between Time, Group, and Optimism on Negative Emotions

**Table S2.** The interaction effect between Time, Group, and Optimism on Positive Emotions

| **Variables** | ***Estimate*** | ***t*** | ***p*** |
| --- | --- | --- | --- |
| Intercept | 23.17 | 6.49 | < .001 |
| Group | -2.68 | -1.66 | .097 |
| Time | -2.92 | -1.36 | .175 |
| Optimism | 0.23 | 0.93 | .351 |
| Group*Time | 2.23 | 2.40 | .017 |
| Group* Optimism | 0.21 | 1.92 | .055 |
| Time* Optimism | 0.35 | 2.40 | .017 |
| Group*Time* Optimism | -0.17 | -2.64 | .009 |

**Table S3.** Differences in the level of Positive Emotions across time for each condition in the case of participants with Low Optimism (below one standard deviation)

| ***Positive Condition*** | | | | ***Ambiguous + Negative Condition*** | | | ***No expectancy Condition*** | | |
| --- | --- | --- | --- | --- | --- | --- | --- | --- | --- |
| ***Time 1 vs Time 2 & Time 3*** | | | | ***Time 1 vs Time 2 & Time 3*** | | | ***Time 1 vs Time 2 & Time 3*** | | |
| **Variables** | Estimate | *t* | *p* | Estimate | *t* | *p* | Estimate | *t* | *p* |
| **Intercept T1** | 23.82 | 21.89 | < .001 | 22.62 | 13.39 | < .001 | 23.66 | 15.66 | < .001 |
| **Time 2** | 3.18 | 1.45 | .149 | 14.35 | 2.41 | .018 | 0.66 | 0.23 | .814 |
| **Time 3** | -1.84 | -0.87 | .384 | -9.79 | -1.56 | .121 | 5.63 | 1.71 | .088 |
| ***Time 2 vs Time 3*** | | | | ***Time 2 vs Time 3*** | | | ***Time 2 vs Time 3*** | | |
| **Variables** | Estimate | *t* | *p* | Estimate | *t* | *p* | Estimate | *t* | *p* |
| **Intercept T2** | 27.16 | 16.64 | < .001 | 36.34 | 2.74 | .007 | 24.48 | 8.85 | < .001 |
| **Time 3** | -5.35 | -2.42 | .016 | -24.38 | -1.72 | .088 | 5.12 | 1.15 | .251 |

*Note.* T**ime 1** – pre-intervention**; Time 2 *–*** post-intervention**; Time 3 *–*** follow-up

**Table S4.** Pairwise comparisons for Positive Emotions in the case of Low Optimism

| ***Time 2, group 1 vs group 2 & group 3*** | | | |
| --- | --- | --- | --- |
| Variables | Estimate | *t* | *p* |
| Intercept G1 | 26.97 | 14.10 | < .001 |
| G 2 | 11.17 | 1.76 | .081 |
| G 3 | -2.61 | -0.98 | .329 |
| ***Time 2, group 2 vs group 3*** | | | |
| Variables | Estimate | *t* | *p* |
| Intercept G2 | 37.79 | 3.91 | < .001 |
| G 3 | -13.06 | -1.29 | .201 |
| ***Time 3, group 1 vs group 2 & group 3*** | | | |
| Variables | Estimate | *t* | *p* |
| Intercept G1 | 22.11 | 8.01 | < .001 |
| G 2 | -9.16 | -0.94 | .349 |
| G 3 | 6.42 | 1.40 | .163 |
| ***Time 3, group 2 vs group 3*** | | | |
| Variables | Estimate | *t* | *p* |
| Intercept G2 | 9.22 | 0.54 | .593 |
| G 3 | 19.78 | 1.11 | .270 |

*Note.* **Time 2 *–*** post-intervention***;* Time 3 *–***follow-up, **Group 1** – Positive Condition (G1), **Group 2** – Ambiguous + Negative Condition (G2) and **Group 3** – No expectancy Condition (G3).

**Table S5.** Differences in the level of Positive Emotions across time for each condition in the case of participants with Medium Optimism

| ***Positive Condition*** | | | | ***Ambiguous + Negative Condition*** | | | ***No expectancy Condition*** | | |
| --- | --- | --- | --- | --- | --- | --- | --- | --- | --- |
| ***Time 1 vs Time 2 & Time 3*** | | | | ***Time 1 vs Time 2 & Time 3*** | | | ***Time 1 vs Time 2 & Time 3*** | | |
| **Variables** | Estimate | *t* | *p* | Estimate | *t* | *p* | Estimate | *t* | *p* |
| **Intercept T1** | 28.07 | 40.55 | < .001 | 28.18 | 46.99 | < .001 | 29.00 | 39.39 | < .001 |
| **Time 2** | 0.68 | 0.59 | .555 | 0.46 | 0.41 | .682 | 1.50 | 1.29 | .197 |
| **Time 3** | 5.02 | 3.73 | < .001 | 4.24 | 3.39 | .001 | 4.85 | 3.74 | < .001 |
| ***Time 2 vs Time 3*** | | | | ***Time 2 vs Time 3*** | | | ***Time 2 vs Time 3*** | | |
| **Variables** | Estimate | *t* | *p* | Estimate | *t* | *p* | Estimate | *t* | *p* |
| **Intercept T2** | 28.76 | 29.52 | < .001 | 28.86 | 28.48 | < .001 | 30.41 | 26.10 | < .001 |
| **Time 3** | 4.57 | 3.28 | .001 | 4.16 | 3.43 | .001 | 3.35 | 2.59 | .010 |

*Note.* T**ime 1** – pre-intervention**; Time 2 *–*** post-intervention**; Time 3 *–*** follow-up.

**Table S6.** Pairwise comparisons for Positive Emotions in the case of Medium Optimism

| ***Time 2, group 1 vs group 2 & group 3*** | | | |
| --- | --- | --- | --- |
| Variables | Estimate | *t* | *p* |
| Intercept G1 | 28.66 | 29.24 | < .001 |
| G 2 | 0.25 | 0.17 | .867 |
| G 3 | 1.89 | 1.40 | .162 |
| ***Time 2, group 2 vs group 3*** | | | |
| Variables | Estimate | *t* | *p* |
| Intercept G2 | 28.91 | 26.39 | < .001 |
| G 3 | 1.44 | 0.94 | .351 |
| ***Time 3, group 1 vs group 2 & group 3*** | | | |
| Variables | Estimate | *t* | *p* |
| Intercept G1 | 33.00 | 23.68 | < .001 |
| G 2 | -0.32 | -0.15 | .884 |
| G 3 | 0.73 | 0.36 | .719 |
| ***Time 3, group 2 vs group 3*** | | | |
| Variables | Estimate | *t* | *p* |
| Intercept G2 | 32.70 | 19.34 | < .001 |
| G 3 | 0.95 | 0.40 | .693 |

*Note.* **Time 2 *–*** post-intervention***;* Time 3 *–***follow-up, **Group 1** – Positive Condition (G1), **Group 2** – Ambiguous + Negative Condition (G2) and **Group 3** – No expectancy Condition (G3).

**Table S7.** Differences in the level of Positive Emotions across time for each condition in the case of participants with High Optimism (over one standard deviation)

| ***Positive Condition*** | | | | ***Ambiguous + Negative Condition*** | | | ***No expectancy*** ***Condition*** | | |
| --- | --- | --- | --- | --- | --- | --- | --- | --- | --- |
| ***Time 1 vs Time 2 & Time 3*** | | | | ***Time 1 vs Time 2 & Time 3*** | | | ***Time 1 vs Time 2 & Time 3*** | | |
| **Variables** | Estimate | *t* | *p* | Estimate | *t* | *p* | Estimate | *t* | *p* |
| **Intercept T1** | 35.30 | 25.00 | < .001 | 33.52 | 19.22 | < .001 | 33.91 | 32.30 | < .001 |
| **Time 2** | 1.46 | 0.41 | .682 | -0.40 | -0.12 | .902 | -3.20 | -2.76 | .006 |
| **Time 3** | 8.33 | 2.09 | .038 | 1.72 | 0.50 | .619 | 0.52 | 0.37 | .715 |
| ***Time 2 vs Time 3*** | | | | ***Time 2 vs Time 3*** | | | ***Time 2 vs Time 3*** | | |
| **Variables** | Estimate | *t* | *p* | Estimate | *t* | *p* | Estimate | *t* | *p* |
| **Intercept T2** | 36.69 | 12.74 | < .001 | 33.77 | 9.77 | < .001 | 31.72 | 24.48 | < .001 |
| **Time 3** | 7.87 | 3.06 | .003 | 2.35 | 0.51 | .613 | 4.34 | 2.74 | .007 |

*Note.* T**ime 1** – pre-intervention**; Time 2 *–*** post-intervention**; Time 3 *–*** follow-up.

**Table S8.** Pairwise comparisons for Positive Emotions in the case of High Optimism

| ***Time 2, group 1 vs group 2 & group 3*** | | | |
| --- | --- | --- | --- |
| Variables | Estimate | *t* | *p* |
| Intercept G1 | 37.02 | 15.30 | < .001 |
| G 2 | -2.78 | -0.95 | .345 |
| G 3 | -5.28 | -1.83 | .069 |
| ***Time 2, group 2 vs group 3*** | | | |
| Variables | Estimate | *t* | *p* |
| Intercept G2 | 34.00 | 15.69 | < .001 |
| G 3 | -2.16 | -0.87 | .383 |
| ***Time 3, group 1 vs group 2 & group 3*** | | | |
| Variables | Estimate | *t* | *p* |
| Intercept G1 | 44.45 | 12.10 | < .001 |
| G 2 | -8.74 | -1.92 | .057 |
| G 3 | -8.08 | -1.84 | .068 |
| ***Time 3, group 2 vs group 3*** | | | |
| Variables | Estimate | *t* | *p* |
| Intercept G2 | 35.90 | 9.72 | < .001 |
| G 3 | 0.58 | 0.13 | .894 |

*Note.* **Time 2 *–*** post-intervention***;* Time 3 *–***follow-up, **Group 1** – Positive Condition (G1), **Group 2** – Ambiguous + Negative Condition (G2) and **Group 3** – No expectancy Condition (G3).
