# Supplementary material for: A randomized trial investigating the impact of response expectancy on the counting blessings intervention: the role of optimism as a moderator
Source: Front Psychol. 2024 Dec 3;15:1399425. doi: 10.3389/fpsyg.2024.1399425 (PMC11649415; doi:10.3389/fpsyg.2024.1399425)
Supplement: Supplementary file 4 [file Data_Sheet_4.docx]

***Appendix:* *Treatment credibility***

**Intervention evaluation form ***

Please indicate below how much you believe, right now, that the intervention based on counting the positive aspects of life helped you. Please answer the questions below in terms of what you think occurred.

**Set I**

1. At this point, how logical do you THINK the intervention based on *counting the positive aspects of life* technique was?

1 2 3 4 5 6 7 8 9

not at all logical somewhat logical very logical

2. At this point, how successfully do you THINK the intervention was in reducing negative emotions?

1 2 3 4 5 6 7 8 9

not at all useful somewhat useful very useful

3. At this point, how successful do you THINK the intervention was in increasing positive emotions?

1 2 3 4 5 6 7 8 9

not at all useful somewhat useful very useful

4. How confident would you be in recommending this intervention to a friend?

1 2 3 4 5 6 7 8 9

not at all confident somewhat confident very confident

*In the original scale by Devilly & Borkovec (2000), we added a new item to the credibility subscale. This was done to balance the perceived effectiveness of the intervention with its impact on both negative and positive emotions.
